# Glial fibrillary acidic protein antibody-associated astrocytopathy presents isolated spinal cord enhancement: A case report

**DOI:** 10.1097/MD.0000000000046689

**Published:** 2026-01-02

**Authors:** Chunxiang Wang, Xin Zou, Xiaoyan Du, Yuan Xue, Weichao Bao, Ying Guo

**Affiliations:** a School of Clinical Medicine, Shandong Second Medical University, Affiliated Hospital of Shandong Second Medical University, Weifang, Shandong Province, China; b Gaomi City Hospital, Gaomi, Weifang, Shandong, China; c Junbukou Town Health Center, Weicheng District, Weifang, Shandong, China; d Department of Endocrinology and Metabolism, The Affiliated Hospital of Shandong Second Medical University, Shandong Second Medical University, Kuiwen District, Weifang City, Shandong Province, China.

**Keywords:** cerebrospinal fluid antibody detection, diabetes mellitus, GFAP antibody-associated astrocytopathy, *Mycoplasma pneumoniae* infection, spinal cord magnetic resonance imaging

## Abstract

**Rationale:**

Glial fibrillary acidic protein antibody–associated astrocytopathy (GFAP-A) is an autoimmune central nervous system inflammatory disorder diagnosed via cerebrospinal fluid (CSF) glial fibrillary acidic protein-immunoglobulin G detection. Clinically, it typically presents with meningoencephalitis or myelitis, accompanied by periventricular perivascular enhancement on brain magnetic resonance imaging (MRI) (findings that guide most routine diagnoses). Yet isolated spinal cord abnormalities (with negative brain MRI) in GFAP-A remain rarely reported, especially when occurring in patients with comorbidities like diabetes mellitus and active infection; these overlapping conditions often mask GFAP-A’s typical features, consequently increasing the risk of clinical misdiagnosis. By reporting this atypical case, the study aims to supplement the existing imaging spectrum of GFAP-A and provide practical diagnostic references for complex clinical scenarios where underlying diseases obscure classic GFAP-A manifestations. It further carries significance in emphasizing the critical role of CSF GFAP-immunoglobulin G detection: this biomarker enables accurate identification of such atypical cases, directly addressing the misdiagnosis risk highlighted earlier.

**Patient concerns:**

A 47-year-old Chinese male patient with a 13-year history of type 2 diabetes mellitus was admitted to the hospital due to fever and headache. Initially, the patient was diagnosed with upper respiratory tract infection based on positive *Mycoplasma pneumoniae* and parainfluenza virus antibodies; however, his symptoms persisted and worsened after treatment with moxifloxacin combined with oseltamivir.

**Diagnoses:**

Emergency CSF examination showed increased pressure, elevated protein level, and monocytosis. Subsequent examination results revealed positive glial fibrillary acidic protein antibodies in the CSF; combined with lumbar MRI showing linear enhancement on the surface of the cauda equina nerves (and negative brain MRI findings), the patient was diagnosed with GFAP-A.

**Interventions:**

The patient received anti-infection treatment, intracranial pressure reduction, and blood glucose control.

**Outcomes:**

After receiving the treatment, the patient’s symptoms improved and he was discharged. At the 2-month follow-up after discharge, the patient still had postural tremor in both upper limbs, but no pathological reflexes were elicited.

**Lessons:**

This case confirms that GFAP-A can be detected with positive spinal cord MRI findings alone, while negative brain MRI findings. Its key clinical significance lies in emphasizing the diagnostic value of CSF glial fibrillary acidic protein antibody detection for atypical cases, providing a new reference for the imaging spectrum of GFAP-A.

## 1. Introduction

Glial fibrillary acidic protein antibody-associated astrocytopathy (GFAP-A) is an autoimmune inflammatory disease of the central nervous system (CNS). Its pathogenesis is closely related to the body’s autoimmune response against glial fibrillary acidic protein (GFAP), which in turn leads to extensive inflammatory damage to the CNS. The clinical manifestations of this disease are extremely diverse, and the lesion involvement is extensive, ranging from the optic nerve to different regions of the spinal cord. Among them, meningoencephalitis is the most common. Currently, diagnosis mainly relies on the detection of GFAP–immunoglobulin G (IgG) antibodies in the cerebrospinal fluid (CSF).

Imaging plays an important and indispensable role in the diagnosis of GFAP-A. According to the study by Fang et al, 75% of patients have diffuse T2-weighted imaging (T2WI) abnormalities in the periventricular white matter; 50% of patients show obvious radial perivascular enhancement; 33% of patients have leptomeningeal enhancement. Spinal cord magnetic resonance imaging (MRI) shows that 71% of patients have extensive longitudinal T2WI hyperintensities; 29% of patients have normal examination results but present with myelitis symptoms.^[1]^ In GFAP-A, lesions can also occur in the basal ganglia, thalamus, brainstem, and cerebellum. Most of these lesions are punctate, showing isointensity or mild hyperintensity on T2WI sequences with blurred boundaries, and perivascular punctate enhancement can be seen.^[2]^

The core difference between this case and previous GFAP-A cases with spinal cord involvement is that in previous cases, spinal cord lesions were almost always accompanied by brain lesions (such as parenchymal abnormalities, perivascular enhancement, etc), while in this case, no brain abnormalities were found on detailed brain MRI, and only linear enhancement on the surface of the cauda equina nerves was shown on lumbar MRI, indicating isolated spinal cord involvement. This manifestation has not been fully reported, which supplements the imaging heterogeneity of GFAP-A and also suggests that clinicians should not rule out this disease due to negative brain MRI findings, but should comprehensively judge based on CSF antibody detection and spinal cord MRI.Herein, we report a GFAP-A patient with 13-year type 2 diabetes mellitus and multiple infections, who presented with isolated spinal cord enhancement, to provide insights for disease identification in complex clinical scenarios.

## 2. Case presentation

The patient was a 47-year-old male with a 13-year history of type 2 diabetes mellitus (T2DM) (complicated with peripheral neuropathy and bilateral stage 2 diabetic retinopathy), grade 3 hypertension, and obesity (BMI 32.1 kg/m²). One week prior to admission, he developed fever (maximum temperature 39.3 °C) accompanied by dizziness, persistent distending pain in the right temporal region (the patient reported “headache worsens with fever and cannot sleep at night due to pain”), cough with white sputum, fatigue, and decreased appetite. Self-administration of nonsteroidal anti-inflammatory drugs (NSAIDs) had poor antipyretic effect, and he sought further treatment in our hospital “for fear of affecting work and life.” On the day of admission, his body temperature was 38.5 °C, which decreased after administration of NSAIDs; he had a history of T2DM (poor blood glucose control), and brain MRI on admission showed lacunar infarction.

On the 2nd day after admission, the patient’s body temperature rose to 38.4 °C and he developed urinary retention (the patient reported “difficulty in urinating and thin urine stream when going to the toilet”); his temperature dropped to 36.3 °C after NSAID administration. After admission, his temperature fluctuated, accompanied by dizziness and headache. Pathogen detection showed positive *Mycoplasma pneumoniae* immunoglobulin M (IgM), positive parainfluenza virus (types 1–3) IgM (indicating possible active infection), Epstein-Barr virus (EBV) viral capsid antigen IgG 67.8 AU/mL (normal range: 0–2 AU/mL), EBV nuclear antigen IgG 89.34 AU/mL (normal range: 0–2 AU/mL), and cytomegalovirus (CMV) IgG 4.55 AU/mL (normal range: <4.2 AU/mL) (IgM was negative for all 3, indicating previous infection). Treatment with moxifloxacin and oseltamivir for anti-infection had poor efficacy. On the 4th day after admission, the patient’s temperature was 37.9 °C, and he reported “aggravated dizziness and headache, sudden inability to speak, and numbness in the right arm” (relieved after 20 minutes). On the same night, he developed slurred speech and slow response again, and emergency brain MRI showed no abnormalities.

On the 5th day after admission, the patient had chills and fever (maximum temperature 38.4 °C) and reported “restlessness and inability to lie still.” Despite administration of chlorpromazine, involuntary movements still occurred, and emergency lumbar puncture was performed. CSF examination results were as follows: pressure > 330 mm H_2_O (normal range: 70–180 mm H_2_O), protein 1.81 g/L (normal range: 0–0.4 g/L), glucose 3.51 mmol/L (normal range: 2.5–4.4 mmol/L), yellowish appearance, cell count 218 × 10⁶/L (normal range: 0–5 × 10⁶/L) with 75% monocytes, IgG 167 mg/L (normal range: 0–34 mg/L), immunoglobulin A (IgA) 36.4 mg/L (normal range: 0–5 mg/L), IgM 2.4 mg/L (normal range: 0–1.3 mg/L), and no tumor cells were found. The simultaneous increase in IgG, IgA, and IgM indicated increased blood–brain barrier (BBB) permeability, further confirming BBB damage.^[3]^ Mannitol was administered to reduce intracranial pressure, and acyclovir was tentatively given for antiviral treatment considering intracranial infection.

On the 6th day after admission, the patient’s consciousness improved, and he could answer questions but remained drowsy. Neurological examination showed positive nuchal rigidity and positive bilateral Kernig sign. The etiology was still unclear. In the following days, the patient remained drowsy but could speak normally, and dizziness persisted. On the 9th day after admission, due to poor blood glucose control, premixed insulin was discontinued, and the patient was switched to subcutaneous injection of 12 units of insulin glargine every night. On the 10th day after admission, the patient reported “dizziness when sitting, body temperature 37.8 °C at night, and poor appetite”; reexamination of inflammatory indicators showed C-reactive protein 11.34 mg/L (normal range: <10 mg/L), monocyte percentage 12.8% (normal range: 3–10%), and serum amyloid A (SAA) 13.41 mg/L (normal range: <10 mg/L), suggesting chronic inflammation. Urethral secretions showed positive *N gonorrhoeae* DNA, and 1 g of ceftriaxone was administered intramuscularly for anti-infection. The results of CSF cell-based indirect immunofluorescence assay detection (collected on the 5th day after admission, results obtained on the 10th day) showed: positive GFAP-IgG antibody (titer 1:32, normal reference value: negative, titer < 1:10; Fig. 1), and negative results for other autoimmune encephalitis antibodies such as N-methyl-D-aspartate receptor, aquaporin 4, and leucine-rich glioma inactivated 1.

**Figure 1.**
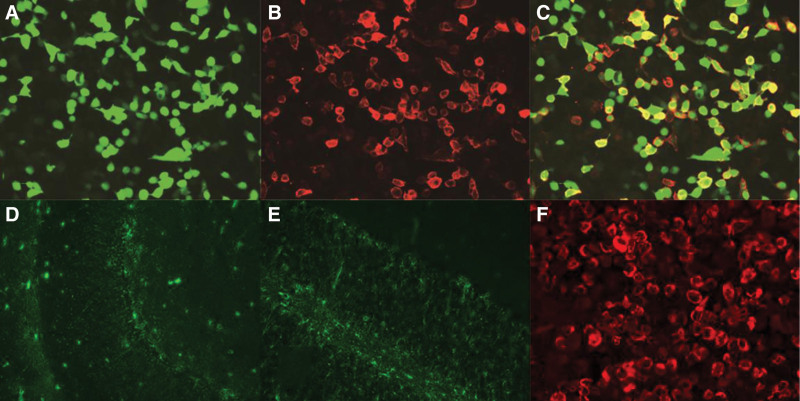
Detection of CSF GFAP–IgG and localization of brain region targets in the patient. (A) Green channel imaging: Transfected cells show green fluorescence; (B) red channel imaging: the cell membrane of transfected cells shows obvious red fluorescence; (C) red-green fluorescence overlap image: no red-green fluorescence co-localization signal was observed in the patient’s sample; (D) hippocampal tissue imaging: astrocytes show endogenous GFAP fluorescence; (E) cerebellar tissue imaging: Astrocytes show endogenous GFAP fluorescence; (F) patient CSF-astrocyte binding imaging: Specific red fluorescence is shown. Cerebrospinal fluid (CSF) collected from the patient on the 5th day after admission was tested for GFAP–IgG antibody using cell-based indirect immunofluorescence assay (CBA).^[3]^ Each panel was observed under a fluorescence microscope: under the green channel (A), if transfected cells show green fluorescence, it indicates successful plasmid transfection; Under the red channel (B), if the cell membrane of transfected cells shows obvious red fluorescence, the antibody is determined to be positive; Under the red-green channel fluorescence overlap observation (C), if there is no red-green fluorescence co-localization signal, nonspecific binding interference is excluded. The patient’s test results showed that: Endogenous GFAP fluorescence of astrocytes was observed in both the hippocampal region (D) and the cerebellar region (E), suggesting that astrocytes in these brain regions can serve as physiological binding targets for GFAP–IgG antibodies; after the patient’s CSF was combined with astrocytes (F), specific red fluorescence was shown, confirming that the GFAP-IgG antibody in the patient’s CSF was positive with a titer of 1:32 (normal reference value: negative, titer < 1:10), and other autoimmune encephalitis antibodies (such as N-methyl-D-aspartate receptor (NMDAR), aquaporin 4 (AQP4), leucine-rich glioma inactivated 1 (LGI1), etc) were negative. CNS = central nervous system; GFAP = glial fibrillary acidic protein; IgG = immunoglobulin G.

Based on the positive GFAP-IgG antibody in the CSF, the patient was considered to have GFAP-A. Edaravone (30 mg) was given orally 3 times a day to improve dizziness. Since the blood glucose did not reach the control target and considering the impact of hormone therapy, the dose of insulin glargine was adjusted to 16 units subcutaneously every night, and insulin aspart was added: 7 units subcutaneously before breakfast, 7 units before lunch, and 5 units before dinner.

**Figure 2.**
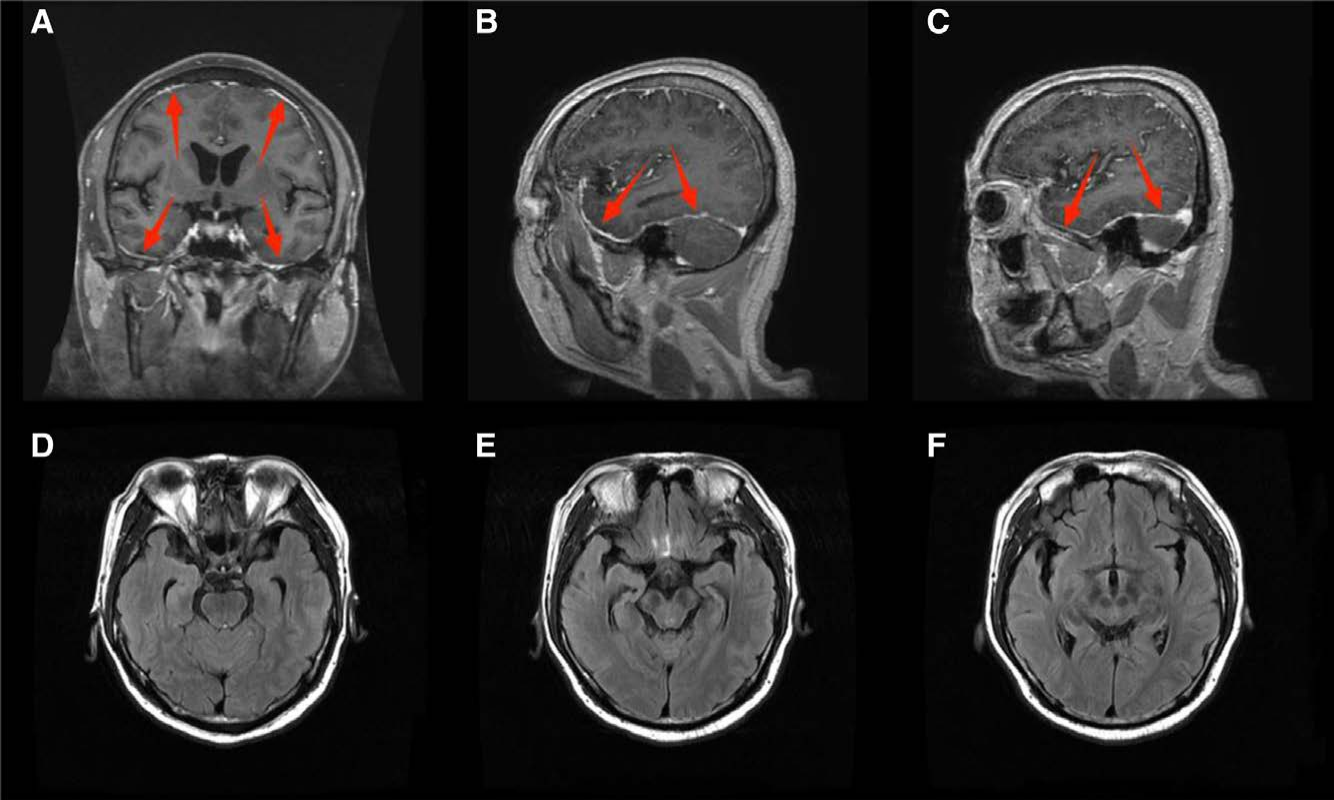
Brain MRI. (A–C) Sagittal and coronal T1-weighted imaging (T1WI) with fat suppression: the surface of the bilateral tentorium cerebelli and dura mater shows mild thickening and rough edges; the areas pointed by red arrows are the thickened and rough parts; (D–F) Axial T2-weighted fluid-attenuated inversion recovery (T2FLAIR) images: no abnormal signals were observed in the bilateral temporal lobes, hippocampus, and insular cortex. MRI = magnetic resonance imaging.

**Figure 3.**
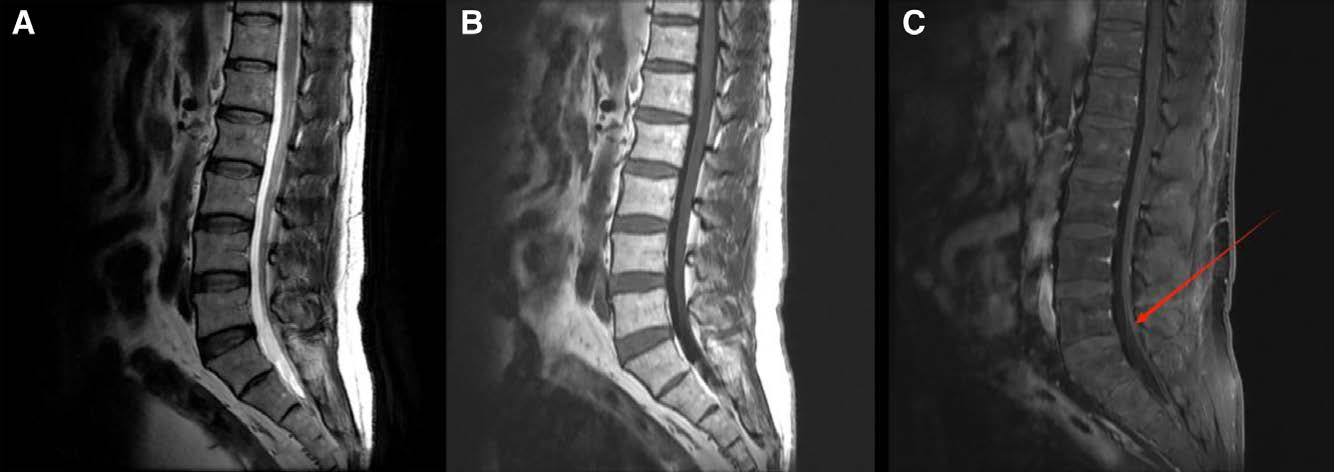
Lumbar MRI. (A) Sagittal T1-weighted imaging (T1WI) of lumbar spine plain scan: no abnormal signals were observed in the conus medullaris and cauda equina nerves; (B) sagittal T2-weighted imaging (T2WI) of lumbar spine plain scan: no abnormal signals were observed in the conus medullaris and cauda equina nerves; (C) sagittal T1WI with fat suppression of lumbar spine enhanced scan: linear enhancement was observed on the surface of the cauda equina nerves; the area pointed by the red arrow is the specific enhanced part. MRI = magnetic resonance imaging.

On the 13th day after admission, the patient had mild nuchal resistance and positive bilateral Kernig sign. Repeated CSF reexamination showed: pressure > 330 mm H_2_O (normal range: 70–180 mm H_2_O), protein 1.82 g/L (normal range: 0–0.4 g/L), cell count 150 × 10^6^/L (normal range: 0–5 × 10^6^/L) with 90% monocytes, glucose 5.3 mmol/L (normal range: 2.5–4.4 mmol/L), chloride 114.1 mmol/L (normal range: 120–130 mmol/L), and negative *Mycobacterium tuberculosis* antibody. Although the positive CSF GFAP–IgG on the 5th day after admission had suggested the possibility of GFAP-A, the significantly decreased CSF chloride (114.1 mmol/L) in the patient was consistent with the CSF characteristics of tuberculous meningitis.^[4]^ Given that concurrent infection may exacerbate GFAP-A-related inflammation and interfere with treatment response, we initiated antituberculosis treatment alongside GFAP-A-targeted therapy to rule out the superimposed effect of tuberculosis on the patient’s condition. Isoniazid (0.6 g/d) and rifampicin (0.45 g/d) were given for antituberculosis, 10 mg of dexamethasone was administered intravenously for anti-inflammation, and mannitol (250 mL q12h) and furosemide (20 mg q12h) were used to strengthen intracranial pressure reduction. Repeated brain MRI showed no typical parenchymal/perivascular enhancement of GFAP-A (only mild thickening and roughness of the bilateral tentorium cerebelli and dura mater, Fig. 2), while lumbar MRI showed linear enhancement on the surface of the cauda equina nerves (Fig. 3).

Combined with the patient’s brain and lumbar MRI findings, CSF results, and clinical manifestations, we diagnosed the patient with GFAP-A. On the 17th day after admission, the patient’s fasting blood glucose was 6.21 mmol/L, and the dose of insulin glargine was adjusted to 23 units subcutaneously before bedtime. On the 19th day after admission, the patient’s serum potassium was 3.34 mmol/L (below the normal range of 3.5–5.5 mmol/L), which was considered related to potassium loss caused by hormone use, and potassium chloride was supplemented. In addition, urethral secretion culture showed positive *N gonorrhoeae* DNA, and the dose of ceftriaxone was adjusted to 2.0 g intravenously every 12 hours. On the 20th day after admission, due to elevated blood glucose caused by glucocorticoid application, the dose of insulin glargine was increased to 28 units subcutaneously before bedtime. On the 25th day after admission, the patient’s drowsiness improved, and metformin 0.5 g was added orally twice a day to strengthen blood glucose control. On the 26th day after admission, lumbar puncture showed: CSF pressure decreased to 250 mm H_2_O (normal range: 70–180 mmH₂O), cell count decreased to 40 × 10^6^/L (normal range: 0–5 × 10^6^/L) with 90% monocytes, and protein decreased to 1.25 g/L (normal range: 0–0.4 g/L). Other indicators were not shown, suggesting improvement in intracranial inflammation and high intracranial pressure symptoms. Therefore, mannitol was discontinued, and a combined intracranial pressure reduction regimen was adopted: furosemide 20 mg orally once a day; spironolactone 20 mg orally once a day; acetazolamide 0.25 g orally twice a day. On the 31st day after admission, dexamethasone was adjusted to prednisone 20 mg orally, and the insulin regimen was simultaneously adjusted to 32 units of insulin glargine subcutaneously before bedtime. On the 36th day after admission, the patient’s fasting blood glucose was 11.3 mmol/L, and 2-hour postprandial blood glucose was 12.9 mmol/L; the dose of insulin glargine was increased to 38 units subcutaneously before bedtime, and the dose of insulin aspart remained unchanged (7 units before breakfast, 7 units before lunch, and 5 units before dinner). On the 39th day after admission, to strengthen postprandial blood glucose control, the insulin regimen was adjusted to 38 units of insulin glargine subcutaneously before bedtime, combined with insulin aspart (9 units before breakfast, 9 units before lunch, and 7 units before dinner). From the 40th day after admission to discharge (43rd day after admission), isoniazid 0.6 g once a day combined with rifampicin 0.45 g once a day was continued for antituberculosis treatment, and the dose of prednisone was gradually reduced. Postprandial blood glucose was monitored to fluctuate between 10 to 12 mmol/L, and the patient had no risk of hypoglycemia. To balance efficacy and insulin dosage, the hypoglycemic regimen was changed to 38 units of insulin glargine subcutaneously before bedtime, combined with insulin aspart (7 units before breakfast, 7 units before lunch, and 5 units before dinner).

At the 2-month follow-up after discharge, the patient’s body temperature was 36.3 °C, pulse 84 beats/min, respiration 18 breaths/min, blood pressure 139/93 mm Hg. He had postural and action tremor in both upper limbs, which was more obvious in the right upper limb. The neck was soft, muscle strength of all limbs was grade 5, muscle tone was normal, and no pathological reflexes were elicited. The patient reported “no major problems with daily life, but tremor still occurs when holding things with both upper limbs” and stated that he had controlled his blood glucose in accordance with medical advice and was confident in subsequent rehabilitation. Reexamination of CSF showed: pressure 140 mm H₂O (normal range: 70–180 mm H_2_O), which had returned to normal; colorless appearance, consistent with normal appearance; chloride concentration 124.4 mmol/L (normal range: 120–130 mmol/L), which was also within the normal range (all these indicators showed significant improvement compared with previous results). The cell count was 18 × 10⁶/L (normal range: 0–5 × 10⁶/L), which was still higher than normal but decreased compared with before; monocytes accounted for 90%, which was unchanged from before; glucose was 6.64 mmol/L (normal range: 2.50–4.45 mmol/L) and protein was 1.710 g/L (normal range: 0–0.4 g/L)) both were still higher than normal. *M tuberculosis* DNA quantitative detection and antibody detection were negative, consistent with previous results, so the diagnosis of tuberculous meningitis was not supported, and antituberculosis drugs were discontinued, with a total antituberculosis treatment course of approximately 82 days. Considering the patient’s condition, the dose of prednisone was increased to 60 mg orally once a day to strengthen immunosuppression for controlling central inflammation; oxiracetam 1 g was also added for intravenous infusion once a day to improve brain metabolism, protect nerve cells, and relieve tremor symptoms. The key clinical events, examination results, and treatment adjustments of the patient during the entire diagnosis and treatment process are shown in Table 1.

**Table 1:** Timeline of key clinical events, key examination results, and important treatment adjustments in the patient’s diagnosis and treatment course.

Time point	Key clinical events	Key examination results	Important treatment adjustments
1 week before admission	Fever (maximum temperature 39.3 °C), right temporal headache, dizziness, cough (with white sputum); self-administration of non-steroidal anti-inflammatory drugs (NSAIDs) showed poor antipyretic effect	–	–
Admission day	Body temperature 38.5 °C (decreased after NSAID administration); past medical history: 13-year history of type 2 diabetes mellitus (T2DM) (complicated with neuropathy/retinopathy), grade 3 hypertension, obesity (BMI 32.1 kg/m²)	Fasting blood glucose 6.21 mmol/L (elevated), glycated hemoglobin 6.73% (elevated); brain MRI showed lacunar infarction	Maintained the original hypoglycemic regimen: insulin degludec-aspart (28 U before dinner) + dapagliflozin (10 mg/day) + metformin (1 g per dose, twice a day)
Day 2 after admission	Body temperature rose to 38.4 °C, developed urinary retention; body temperature decreased to 36.3 °C after NSAID administration	–	Discontinued dapagliflozin (to prevent urinary tract infection)
Day 4 after admission	Body temperature 37.9 °C, aggravated dizziness and headache; developed “aphasia + numbness in the right upper limb (relieved after 20 minutes)” and “slurred speech + slow response”; transient ischemic attack (TIA) was suspected	Emergency brain MRI: no abnormalities	No specific treatment was given
Day 5 after admission	Chills, high fever (38.4 °C), restlessness, involuntary movements	Lumbar puncture: cerebrospinal fluid (CSF) pressure > 330 mm H_2_O (significantly elevated), protein 1.81 g/L (significantly elevated), cell count 218 × 10⁶/L (75% monocytes), and elevated IgG/IgA/IgM	Acyclovir for antiviral therapy + mannitol for intracranial pressure reduction + chlorpromazine to relieve restlessness
Day 10 after admission	Dizziness when sitting, low-grade fever (37.8 °C), poor appetite	CSF GFAP-IgG positive (titer 1:32); *Neisseria gonorrhoeae* DNA positive in urethral secretions; elevated C-reactive protein (CRP)/serum amyloid A (SAA)/monocyte percentage	Ceftriaxone (1 g intramuscular injection, for gonorrhea) + idebenone (to improve dizziness); adjusted the hypoglycemic regimen to insulin glargine (16 U/night) + insulin aspart (7 U before breakfast, 7 U before lunch, 5 U before dinner)
Day 13 after admission	Low-grade fever (37.7 °C), nuchal rigidity, positive bilateral Kernig sign	Lumbar MRI showed linear enhancement on the surface of the cauda equina nerves; brain MRI showed no parenchymal/perivascular enhancement; repeated CSF examination remained abnormal	Added isoniazid + rifampicin (for antituberculosis) + dexamethasone (10 mg intravenous infusion, for anti-inflammation); strengthened intracranial pressure reduction (mannitol + furosemide)
Day 26 after admission	Drowsiness improved	Repeated lumbar puncture: CSF pressure 250 mm H_2_O (decreased), cell count 40 × 10⁶/L (decreased), protein 1.25 g/L (decreased)	Discontinued mannitol; switched to oral furosemide + spironolactone + acetazolamide for intracranial pressure reduction
Day 43 after admission (discharge)	Symptoms improved	–	Continued isoniazid + rifampicin; gradually reduced the dose of prednisone; hypoglycemic regimen: insulin glargine (38 U/night) + insulin aspart (7 U before breakfast, 7 U before lunch, 5 U before dinner) + metformin
Two months after discharge (follow-up)	Postural tremor in both upper limbs (more obvious in the right upper limb), normal muscle strength, no pathological signs	Normal CSF pressure, negative tuberculosis-related tests; blood glucose/glycated hemoglobin remained elevated	Discontinued antituberculosis drugs; increased prednisone to 60 mg/day (to strengthen immunosuppression) + oxiracetam (to improve brain metabolism); adjusted the hypoglycemic regimen

## 3. Outcomes

For the patient’s multiple infections, initial treatment included moxifloxacin and oseltamivir. Later, when *N gonorrhoeae* was found, ceftriaxone was used. To control CNS inflammation, dexamethasone was given intravenously for 3 days, then prednisone was taken by mouth. Mannitol helped reduce intracranial pressure, and insulin was used to manage blood glucose.

Based on the aforementioned interventions (anti-infection therapy, immunosuppressive therapy, intracranial pressure reduction, and blood glucose control), the patient’s clinical outcomes from the acute hospitalization phase to the 2-month follow-up after discharge were comprehensively evaluated through dynamic monitoring of clinical symptoms, laboratory indicators, and imaging findings. At admission, the patient presented with persistent high fever (maximum temperature 39.2 °C), prominent headache, dysuria (onset on the 2nd day after admission, persisting until the 34th day when the urinary catheter was removed), transient aphasia (self-resolving within 20 minutes), and numbness of the right upper limb. Following treatment, the high fever resolved 3 days after adjusting the anti-infection regimen (initial moxifloxacin for *M pneumoniae* and oseltamivir for parainfluenza virus, switched to ceftriaxone after detecting *N gonorrhoeae*), with body temperature stabilizing at 36.5 to 37.2 °C; the headache alleviated significantly without the need for additional analgesics; transient aphasia and right upper limb numbness did not recur; and dysuria gradually improved with treatment, eventually leading to successful catheter removal. Throughout the course, neurological examinations consistently showed grade 5 muscle strength in all limbs and no abnormal sensory levels, indicating that dysuria was unrelated to transverse spinal cord injury and might have synergistically occurred with benign prostatic hyperplasia common in middle-aged men. In terms of laboratory indicators, emergency CSF testing at admission revealed marked abnormalities: intracranial pressure 280 mm H_2_O (normal range: 80–180 mm H_2_O), protein level 1.81 g/L (normal range: 0.15–0.45 g/L), monocyte-predominant pleocytosis (218 × 10^6^/L, with monocytes accounting for 75%), and positive CSF GFAP-IgG (titer 1:32). After 2 weeks of treatment, a repeat CSF test showed improvements: intracranial pressure decreased to 160 mm H_2_O, protein level dropped to 0.62 g/L, monocyte count reduced to 40 × 10^6^/L (monocytes accounting for 90%), and CSF GFAP–IgG remained positive. For blood glucose control, fasting blood glucose at admission was 11.3 mmol/L, and after adjusting the insulin regimen, it stabilized at 6.5 to 7.8 mmol/L by discharge. SAA at admission was 13.41 mg/L (normal reference value: <10 mg/L) and decreased to the normal range after treatment, indicating controlled infection-related inflammation. Imaging findings showed linear enhancement on the surface of the cauda equina nerves on lumbar enhanced MRI at admission (Fig. 3C), with no progression on reexamination on the 14th hospital day and no spinal cord parenchymal edema or signal abnormalities. Two brain MRI scans (including T2-weighted fluid-attenuated inversion recovery sequence) showed no periventricular enhancement or parenchymal abnormalities, only mild thickening and roughness of the bilateral tentorium cerebelli and dura mater (Fig. 2A–C), without basal cistern enhancement or tuberculoma manifestations. During the 2-month follow-up after discharge, the patient still had mild postural tremor in both upper limbs, which affected fine movements (e.g., holding a pen) but not daily living activities; no recurrence of fever, headache, or dysuria was observed, and neurological examinations showed no pathological reflexes with grade 5 muscle strength in all limbs. Follow-up laboratory tests revealed a further decrease in CSF protein to 0.51 g/L and monocyte count to 18 × 10^6^/L (near normal), though CSF GFAP-IgG titer was not remeasured; fasting blood glucose remained stable at 6.0 to 7.2 mmol/L, and inflammatory indicators were within the normal range. Lumbar MRI follow-up showed no new lesions, with persistent linear enhancement on the cauda equina nerve surface (no progression), consistent with the chronic inflammatory phase of GFAP-A.

## 4. Discussion

GFAP-A is a type of autoimmune disease characterized by CNS inflammation. Its diagnosis mainly relies on the detection of GFAP–IgG in CSF. Although periventricular white matter perivascular radial enhancement is a common imaging finding, it also has heterogeneous features involving the meninges and spinal cord.^[5]^ This case involved a 47-year-old male patient with a 13-year history of T2DM, complicated with multiple infection factors, and had CSF findings suggestive of tuberculous meningitis. These infection, metabolic, and suspected tuberculosis factors may directly or indirectly cause neurological symptoms. It is necessary to systematically rule out other diseases based on the pathogenesis of GFAP-A and literature evidence to clarify the core pathogenic role of GFAP autoimmune response and avoid diagnostic bias caused by interference from underlying diseases.

When clarifying the pathogenic role of infection-related factors, it is first necessary to determine whether infection directly causes spinal cord lesions (taking postinfectious myelitis [which directly causes spinal cord lesions] as a typical example). It is essentially a spinal cord inflammatory demyelinating disease induced by direct pathogen invasion, with acute spinal cord parenchymal injury as the core pathological change, which is completely inconsistent with the core characteristics of this case. In terms of clinical manifestations, postinfectious myelitis is often accompanied by transverse injury signs below the lesion level, such as paraplegia, loss of sensory level, and urinary and fecal dysfunction.^[6]^ Although this patient developed dysuria from the 2nd day after admission (the symptom persisted until the 34th day when the urinary catheter was removed), neurological examination throughout the course showed grade 5 muscle strength in all limbs without abnormal sensory level, and the dysuria symptom may have a synergistic effect with benign prostatic hyperplasia common in middle-aged men, which cannot be used as specific evidence of transverse spinal cord injury. In terms of imaging, the typical MRI finding of postinfectious myelitis is “long-segment T2-weighted imaging (T2WI) hyperintensity of the spinal cord (involving ≥ 3 vertebral segments) + spinal cord parenchymal edema and thickening,” while the lumbar enhanced MRI of this case only showed linear enhancement on the surface of the cauda equina nerves (Fig. 3C) without abnormal signals or edema changes in the spinal cord parenchyma. This imaging feature is highly consistent with the research conclusion of Fang team, which pointed out that GFAP-A can present with spinal nerve root enhancement, not limited to periventricular radial enhancement.^[1]^

The difference in CSF indicators further supports the differentiation of postinfectious myelitis: the early CSF of postinfectious myelitis is dominated by elevated neutrophils without specific autoantibodies.^[6]^ In this case, 2 CSF tests showed monocyte predominance and positive GFAP-IgG, which is a typical feature of GFAP-A confirmed by Flanagan et al.’s study of 102 patients (they explicitly stated that CSF GFAP-IgG positivity combined with monocytic pleocytosis has extremely high diagnostic specificity for GFAP-A).^[7]^ In this case, the 2 CSF examinations on the 5th and 13th days after admission were dominated by monocytes (accounting for 75% and 90% respectively), and GFAP-IgG was positive (titer 1:32) at the same time. This result is consistent with the conclusion of a clinical study on 102 GFAP-A patients, which clearly stated that CSF GFAP-IgG is a specific biomarker of GFAP-A, and its positivity has extremely high specificity for disease diagnosis.^[7]^ In addition, regarding the patient’s positive EBV and CMV IgG but negative IgM, a study on EBV infection in children (led by Sohn team) revealed that among patients with primary EBV infection, 26.8% will have simultaneous positive EBV and CMV IgG, but only 2.5% of them are true CMV co-infections, and the remaining 97.5% are CMV false positives, which may be related to immune cross-reactivity or nonspecific interference of detection methods.^[8]^ This conclusion can rule out the possibility of active CMV infection in this case, and clarify that the current active infections are mainly *M pneumoniae* and parainfluenza virus.

From the perspective of the mechanism of infection triggering GFAP-A, a prospective study of 90 GFAP-A patients revealed that viral infection is a potential trigger of GFAP-A. The specific mechanism is to activate the immune system through molecular mimicry, induce the production of anti-GFAP antibodies and the activation of GFAP-specific CD8^+^T cells, thereby damaging the BBB and causing T cell-mediated autoimmune damage.^[9]^ A study on the immune response to respiratory viruses confirmed that respiratory viruses such as parainfluenza virus can promote the maturation of dendritic cells by activating the mitochondrial antiviral signaling protein-dependent pathway of respiratory epithelial cells, and ultimately induce the differentiation of naive CD8^+^T cells into effector CD8^+^T cells.^[10]^ A study on childhood mycoplasma pneumonia also found that the proportion of CD8^+^T cells in the peripheral blood of patients with acute *M pneumoniae* infection is significantly higher than that of the healthy control group, and this proportion is positively correlated with the level of SAA, suggesting that the activation degree of CD8^+^T cells is closely related to the intensity of local inflammatory response.^[11]^ The patient’s serum SAA level of 13.41 mg/L (normal reference value < 10 mg/L) in this case exactly confirms this mechanism. In terms of treatment response, the initial anti-infection regimen of this case (moxifloxacin for *M pneumoniae*, oseltamivir for parainfluenza virus, and ceftriaxone for *N gonorrhoeae*) was ineffective, but after adding 10 mg of dexamethasone for intravenous infusion, the CSF cell count decreased from 218 × 10⁶/L to 40 × 10⁶/L, and the inflammatory indicators were significantly relieved, which is consistent with the conclusion reported by Fang team that GFAP-A is sensitive to immunosuppressive therapy.^[1]^ In conclusion, postinfectious myelitis can be clearly ruled out.

In addition to infection factors, the patient’s 13-year history of type 2 diabetes mellitus (long-term poor blood glucose control) may also cause nerve damage through metabolic disorders, so it is necessary to further investigate diabetic-related encephalopathy. This disease is a diffuse brain dysfunction directly caused by hyperglycemia, which is essentially brain damage caused by glucose metabolism disorders. The manifestations of this case are completely inconsistent with the diagnostic points of this disease. In terms of symptom characteristics, diabetic metabolic encephalopathy is characterized by diffuse brain dysfunction such as “confusion, persistent drowsiness, and cognitive decline,” and symptom onset is mostly directly related to hyperglycemic crisis with random blood glucose > 16.7 mmol/L.^[12]^ This case only had transient aphasia (self-relieved after 20 minutes) and numbness of the right upper limb, without diffuse manifestations such as cognitive impairment and consciousness disturbance, and no random blood glucose > 16.7 mmol/L was found in the whole blood glucose monitoring process, and there was no clear temporal correlation between symptom onset and blood glucose fluctuation. In terms of CSF performance, diabetic metabolic encephalopathy shows noninflammatory changes, and CSF protein and immunoglobulin levels are within the normal range. However, in this case, CSF protein was 1.81 g/L (significantly higher than the upper normal limit), and IgG, IgA, and IgM levels increased simultaneously (167 mg/L, 36.4 mg/L, and 2.4 mg/L respectively, all higher than the normal range), suggesting increased BBB permeability. This feature is consistent with the conclusion of the study on the role of CSF immunoglobulins in encephalopathy differentiation.^[3]^ At the same time, GFAP-IgG was positive in this case, showing typical immune-inflammatory changes, which is contrary to the pathological nature of metabolic encephalopathy.

At the mechanism level, a study on diabetes and BBB revealed that diabetes can damage brain microvessels and BBB through oxidative stress and pro-inflammatory effects. Among them, the activation of the receptor for advanced glycation end products will aggravate vascular dysfunction through multiple pathways, and the BSCB is structurally more fragile than the BBB, with lower expression of tight junction proteins and poorer tolerance to hyperglycemia.^[13]^ Prasad et al revealed that diabetes damages the BBB and BSCB through oxidative stress, with lower tight junction protein expression in BSCB making it more vulnerable to hyperglycemia.^[13]^ Consistent with this, Wu et al further confirmed that hyperglycemia aggravates BSCB damage via excessive endothelial ferroptosis, which may explain the isolated spinal cord involvement in our case.^[14]^ This explains the feature of isolated spinal cord involvement in this case, based on the patient’s clinical data and established mechanisms: The patient’s 13-year poorly controlled T2DM damaged the BSCB (known to have weaker tight junction protein expression than the BBB and poorer tolerance to hyperglycemia) while hyperglycemia further exacerbated this damage by triggering excessive ferroptosis of endothelial cells. Meanwhile, active *M pneumoniae*/parainfluenza virus infection activated CD8^+^T cells, which is consistent with the patient’s elevated SAA level of 13.41 mg/L (normal < 10 mg/L). These 2 factors synergistically enabled GFAP-specific immune cells to infiltrate the spinal cord (with no intracranial involvement, as the BBB remained intact), in line with recognized BSCB damage-mediated immune infiltration processes.The preferential damage of BSCB provides a pathway for GFAP autoimmune response, while the negative brain MRI confirms that the BBB has no significant damage, which is consistent with the selective damage mechanism of diabetes to different barriers. Symptoms of diabetic metabolic encephalopathy can be quickly relieved after strict hypoglycemic treatment, but in this case, the hypoglycemic regimen was adjusted many times, but the neurological symptoms did not improve, and the symptoms were relieved only after the intervention of dexamethasone, so diabetic metabolic encephalopathy can be ruled out.

For the suspected tuberculous meningitis on the 13th day after admission, it is necessary to gradually rule out it based on etiological evidence, imaging features, and treatment response. The diagnosis of tuberculous meningitis relies on etiological evidence of *M tuberculosis* infection, such as positive CSF *M tuberculosis* antibody, positive DNA detection, or positive CSF culture. In this case, multiple CSF *M tuberculosis* antibody and DNA detections were negative, and after standardized antituberculosis treatment with isoniazid (0.6 g/d) combined with rifampicin (0.45 g/d) for 82 days, the reexamined brain MRI showed no significant change in dural thickening, and the CSF indicators did not improve specifically. After stopping antituberculosis drugs, the condition did not recur, which lacks etiological and treatment response support for tuberculous meningitis. In terms of imaging, tuberculous meningitis is often accompanied by typical manifestations such as basal cistern enhancement and cerebral parenchymal tuberculomas. The brain MRI of this case only showed mild thickening and roughness of the bilateral tentorium cerebelli and dura mater (Fig. 2A–C), without basal cistern enhancement or tuberculoma changes. This dural manifestation (mild thickening of the tentorium cerebelli) is consistent with Ke et al’s observation that GFAP-A can present with heterogeneous meningeal enhancement patterns beyond classic periventricular changes.^[2]^ Flanagan et al’s study also indirectly supports this, as they noted that GFAP-A’s inflammatory involvement is not limited to cerebral parenchyma.^[7]^ After excluding the patient’s history of malignant tumors, no dural space-occupying lesions were found on brain MRI, CSF tumor cytology was negative, and no bacteria or fungi were grown in blood and CSF cultures, so dural reactions related to tumors and other infections can be ruled out, and it is clear that this dural change is an atypical manifestation of GFAP-A. In terms of treatment, the inflammatory indicators of tuberculous meningitis will gradually ease after standardized antituberculosis treatment. The decrease in CSF cell count and protein level in this case all occurred after the addition of dexamethasone, which has no direct correlation with antituberculosis drugs, further confirming that the diagnosis of tuberculous meningitis is not established.

After excluding the above diseases, the core features of this case (positive CSF GFAP-IgG [titer 1:32], linear enhancement on the surface of the cauda equina nerves shown by lumbar MRI, and effective immunosuppressive therapy) are related to the research conclusion of Ke team on 34 GFAP-A patients. The study confirmed that spinal cord involvement in GFAP-A has significant heterogeneity: 70.4% of patients with spinal cord lesions present with long-segment or transverse intramedullary T2WI hyperintensities, meningeal enhancement and spinal central canal enhancement are common enhancement patterns, and 85.3% of patients are sensitive to hormone-based immunosuppressive therapy.^[2]^ The characteristic of this case that “symptoms relieved after hormone treatment” is consistent with the treatment response of GFAP-A in this study, which provides important support for the diagnosis. It should be noted that Ke team’s study did not clearly propose that isolated spinal cord or nerve root enhancement can be used as an atypical imaging manifestation of GFAP-A. Among the 33 patients who underwent brain MRI in the study, 90.9% had abnormal intracranial signals, and no spinal cord-involved subtypes without intracranial lesions were reported.^[5]^ However, combined with the widely confirmed imaging heterogeneity of GFAP-A, the characteristics of this case still conform to the disease spectrum of this disease.

It should be noted that most of the previously reported GFAP-A cases with spinal cord involvement are accompanied by brain lesions (such as parenchymal abnormalities, perivascular enhancement, etc), while in this case, no intracranial abnormalities were found on detailed brain MRI (including T2-weighted fluid-attenuated inversion recovery sequence), and only isolated cauda equina nerve enhancement was observed. This manifestation has not been fully reported so far and has certain clinical particularity. From the perspective of pathogenic mechanism, *M pneumoniae* and parainfluenza virus infection may create a local immune-inflammatory microenvironment by activating CD8^+^T cells, and diabetes may reduce the pathological threshold of immune cell infiltration by damaging the BSCB. Both are more likely to be triggers of GFAP autoimmune response rather than etiologies, and jointly promote the occurrence and development of the disease. This special manifestation of the case may provide new clinical evidence for the imaging heterogeneity of GFAP-A, suggesting that in clinical practice, for patients with neurological symptoms complicated with infection and underlying metabolic diseases, if brain MRI has no typical abnormalities, it is necessary to pay attention to the combined evaluation of CSF GFAP-IgG detection and spinal cord MRI, so as to avoid ignoring the potential diagnosis of GFAP-A due to excessive attention to underlying diseases.

While this case provides valuable insights for identifying atypical GFAP antibody-associated astrocytopathy (GFAP-A) in complex clinical scenarios (e.g., comorbid T2DM and multiple infections), it is important to acknowledge its inherent limitations. First, as a single-case report, the findings lack generalizability to the broader GFAP-A population. The isolated spinal cord enhancement (cauda equina nerve surface linear enhancement) with negative brain MRI observed in this patient has not been widely reported, and it remains unclear whether this represents a rare subtype of GFAP-A or an occasional phenotypic variation; validation through larger, multicenter cohort studies is therefore needed to confirm its prevalence and clinical relevance. Second, the follow-up duration was limited to 2 months, which is insufficient to assess long-term outcomes. Critical data (such as the resolution of bilateral upper limb postural tremor, dynamic changes in spinal cord MRI enhancement (e.g., whether the linear enhancement persists or fades), and fluctuations in CSF GFAP-IgG titer (a key biomarker for monitoring GFAP-A activity) are missing, making it difficult to evaluate disease stability, long-term prognosis, and the sustained efficacy of treatment. Third, the patient’s complex comorbidities (13-year history of poorly controlled T2DM, grade 3 hypertension, and active infections with *M pneumoniae*, parainfluenza virus, and *N gonorrhoeae*) introduce confounding factors. These conditions may have synergistically affected the severity of neurological symptoms (e.g., urinary retention, headache) and responses to anti-infection/immunosuppressive therapy, complicating efforts to isolate the independent pathogenic role of GFAP autoimmunity from the effects of metabolic dysfunction or infection-induced inflammation. Finally, CSF GFAP-IgG titer was not remeasured during the 2-month follow-up. This missing data limits direct evidence of whether the administered therapies (e.g., dexamethasone, insulin) reduced GFAP-specific antibody levels (a critical indicator of targeted efficacy against GFAP-A). These limitations highlight the need for future studies with larger sample sizes and extended follow-up to further refine the understanding of atypical GFAP-A phenotypes and their optimal management strategies.

Based on the special clinical scenario of this case with multiple underlying diseases and multiple infections, its diagnosis and treatment process suggests that for GFAP-A cases in complex backgrounds, the condition changes are complex and the principle of individualized dynamic adjustment should be followed. In terms of infection control, the regimen should be optimized in a timely manner according to pathogen detection results. For example, in this case, moxifloxacin and oseltamivir were initially used for Mycoplasma and viral infections, and ceftriaxone was adjusted for anti-infection after the detection of *N gonorrhoeae*. Immunomodulatory therapy should be dynamically optimized based on inflammatory indicators: first, intravenous pulse therapy with dexamethasone was used to quickly control central inflammation, then oral prednisone was used for maintenance. During follow-up, due to the patient’s persistent upper limb tremor and elevated CSF protein, the dose of prednisone was increased to strengthen immunosuppression. At the same time, blood glucose management should be strengthened simultaneously, and the insulin regimen should be actively adjusted to reduce further damage to BSCB caused by hyperglycemia. In terms of prognostic follow-up, based on the fact that the patient still had elevated CSF cell count (18 × 10⁶/L), significantly elevated protein (1.710 g/L), and postural tremor of both upper limbs 2 months after discharge, it is recommended to extend the follow-up period to more than 2 years, and reexamine 3 core indicators (CSF GFAP-IgG titer, spinal cord MRI, blood glucose-related indicators) every 3 to 6 months. If inflammatory indicators rebound or neurological symptoms worsen during hormone reduction, immunosuppressants such as mycophenolate mofetil can be considered, and liver and kidney functions should be closely monitored to balance the efficacy and safety of treatment.

## 5. Conclusion

In conclusion, CSF GFAP–IgG is not only a key marker for the early diagnosis of GFAP-A, but also its dynamic changes can assist in evaluating the treatment response. The implementation of individualized management and long-term follow-up strategies can not only provide diagnostic and treatment references for similar GFAP-A cases complicated with underlying diseases and infections, but also further highlight the clinical value of antibody detection in the whole-cycle management of the disease.

## Acknowledgments

We acknowledge the support provided by Ms. Yanming Ge, as well as the medical staff of the Department of Neurology and the Department of Medical Imaging at the Affiliated Hospital of Shandong Second Medical University, in clinical diagnosis and treatment assistance, imaging diagnosis support, and case data collation for this study.

## Author contributions

**Investigation:** Yuan Xue.

**Writing – original draft:** Chunxiang Wang.

**Supervision:** Xiaoyan Du.

**Validation:** Weichao Bao.

**Resources:** Xin Zou.

**Writing – review & editing:** Ying Guo.
